# Co-extinctions annihilate planetary life during extreme environmental change

**DOI:** 10.1038/s41598-018-35068-1

**Published:** 2018-11-13

**Authors:** Giovanni Strona, Corey J. A. Bradshaw

**Affiliations:** 1 European Commission, Joint Research Centre, Directorate D – Sustainable Resources, Ispra, Italy; 2 ARC Centre of Excellence for Australian Biodiversity and Heritage, Global Ecology, College of Science and Engineering, Flinders University, Adelaide, Australia

## Abstract

Climate change and human activity are dooming species at an unprecedented rate via a plethora of direct and indirect, often synergic, mechanisms. Among these, primary extinctions driven by environmental change could be just the tip of an enormous extinction iceberg. As our understanding of the importance of ecological interactions in shaping ecosystem identity advances, it is becoming clearer how the disappearance of consumers following the depletion of their resources — a process known as ‘co-extinction’ — is more likely the major driver of biodiversity loss. Although the general relevance of co-extinctions is supported by a sound and robust theoretical background, the challenges in obtaining empirical information about ongoing (and past) co-extinction events complicate the assessment of their relative contributions to the rapid decline of species diversity even in well-known systems, let alone at the global scale. By subjecting a large set of virtual Earths to different trajectories of extreme environmental change (global heating and cooling), and by tracking species loss up to the complete annihilation of all life either accounting or not for co-extinction processes, we show how ecological dependencies amplify the direct effects of environmental change on the collapse of planetary diversity by up to ten times.

## Introduction

Being in the midst of the sixth mass extinction^1^, it is fitting to quantify the relative contribution of different mechanisms driving catastrophic biodiversity loss. Drivers directly related to anthropogenic modifications of the biosphere are apparent and well-described: habitat destruction, over-exploitation, and biotic invasions^2^. Similarly, the effects of environmental change (e.g., temperature rise, increased droughts, ocean acidification, *et cetera*) can be easily interpreted — when the environmental conditions of a certain locality become incompatible with the tolerance limits of inhabiting species, in many cases these will go locally extinct, just like fish in an aquarium with a broken thermostat (even if there are counter examples of species that have been capable of rapid adaptation to novel environmental conditions^3^). Yet, there are other, more complicated mechanisms that can exacerbate species loss. In particular, it is becoming increasingly evident how biotic interactions, in addition to permitting the emergence and maintenance of diversity, also build up complex networks through which the loss of one species can make more species disappear (a process known as ‘co-extinction’), and possibly bring entire systems to an unexpected, sudden regime shift, or even total collapse^4–9^.

In a simplified view, the idea of co-extinction reduces to the obvious conclusion that a consumer cannot survive without its resources. Because resource and consumer interactions in natural systems (e.g., food webs) are organized in various hierarchical levels of complexity (e.g., trophic levels), it follows that the removal of resources could result in the cascading (bottom-up) extinction of several higher-level consumers^8,10^. Several studies based on either simulated or real-world data suggest that we should expect most events of species loss to cause co-extinctions^5^, as corroborated by the worrisome, unnatural rate at which populations and species are now disappearing^11^, and which goes far beyond what one expects as a simple consequence of human endeavour^1^. In fact, even the most resilient species will inevitably fall victim to the synergies among extinction drivers^2^ as extreme stresses drive biological communities to collapse. Furthermore, co-extinctions are often triggered well before the complete loss of an entire species^12^, so that even oscillations in the population size of a species could result in the local disappearance of other species depending on the first^13^.

This makes it difficult to be optimistic about the future of species diversity in the ongoing trajectory of global change, let alone in the case of additional external, planetary-scale catastrophes. A previous study^14^ contended this idea by using the remarkable tolerance of tardigrades to extreme temperature, pressure, and radiation as a reference to calculate the likelihood of global sterilization on an Earth-like planet following different, dramatic astrophysical events. The stunning conclusion of that study is that life on our planet has the potential to survive asteroid impacts, supernovae, and gamma-ray bursts^14^. This ostensibly reassuring news highlights how some scientists still tend to disregard the role of co-extinctions within collapsing communities in driving global biodiversity loss, while focusing on individual species’ tolerance limits as the only criteria relevant to species survival in a changing world. Ecologists know the optimism is not supported quantitatively, but can we estimate the magnitude of the bias?

Here we attempt to do this by combining real-world ecological and environmental data to generate several virtual Earths populated by interconnected species-interaction networks where we allow species to move and adapt, that we then subjected to extreme, global environmental change. By comparing scenarios of extinctions based only on species’ environmental tolerances with others accounting also for co-extinctions, we show that neglecting to consider the cascading effect of biodiversity loss leads to a large overestimation of the robustness of planetary life to global change.

## Results and Discussion

We populated 2000 ‘virtual Earths’ with species-like entities arranged in interconnected ecological communities. We then subjected those Earths to catastrophic environmental change eventually resulting in the annihilation of all planetary life. We randomly assigned species to communities on the basis of their tolerance to local climatic conditions, and then we arranged them into structured food webs that we built by linking resources to consumers under various ecological constraints (e.g., trophic level, consumer specificity, and functional-trait compatibility; see Methods). Before and while applying environmental change to the virtual Earths, we simulated dispersal processes between communities, with the success of colonization contingent on dispersal distance, and on the ability of a potential colonizer to enter the target community by displacing other species through superior competitive ability. This gave some biogeographical and macro-ecological realism to our simulated planet.

To keep our model simple yet realistic, we focused on local temperatures and the thermal tolerances of species to them, limiting our simulations to the terrestrial domain, because marine ecosystems likely experience different susceptibility to incremental changes in temperature given the higher specific heat capacity of water relative to air. Initial temperature of each virtual Earth mimicked that of our planet today, and we assigned species ecologically plausible features (temperature tolerance limits, trophic level, specialization) obtained from empirical datasets (see Methods). We applied two main trajectories of environmental change — a monotonic linear increase in temperatures, or a progressive cooling (e.g., a ‘nuclear winter’) such as that predicted following multiple nuclear detonations^15^ or an asteroid impact^14^. To increase the realism of our simulated trajectories, and to obtain conservative curves of diversity loss relative to incremental changes in temperature, we permitted species to adapt to some extent to changing conditions by extending their tolerance limits in the same direction as the environmental change experienced. Furthermore, we permitted the replenishment of depleted populations through regular immigration of recruits from surrounding areas (see Methods for details).

For both trajectories of environmental change, we tracked the loss of diversity within two, separate scenarios until complete species annihilation. In the first scenario, we followed the logic underlying Sloan and colleagues^14^, by dooming species to extinction only according to their individual environmental tolerances. In the second scenario, we also accounted for co-extinctions^4–10^. This permitted us to quantify, by orders of magnitude, the gap between the diversity loss due to a species’ inability to cope with changing conditions, and that due to the domino effect of co-extinctions following primary extinctions. We assumed that the co-extinction of a consumer could be triggered before the complete depletion of all of its resources, and that resources freed up by the extinction of consumers could be redistributed to some extent to surviving consumers in the network^12^ (see Methods for details).

Throughout the full set of simulations, we randomly varied the parameters controlling the thresholds for the amount of resource depletion triggering a consumer’s co-extinction, and the degree of possible reallocation of unused resources. In this way, we explored a wide range of potential model settings, from optimistic ones regarding the robustness of ecological networks to species loss (with consumers going extinct only after the full depletion of most of their resources, and most resources freed up by the consumers’ extinction reallocated to remaining consumers), to pessimistic ones where small losses in resources triggered co-extinctions, and with little adaptability of the network. Similarly, in each simulation we varied at random other important model parameters controlling the amount of initial species diversity, the number of populated localities in the virtual Earth, and the frequency of colonization events (both before and during environmental change). This allowed us to identify both conservative upper and lower boundaries to our projections, and to investigate the sensitivity of our results to model assumptions.

**Figure 1:**
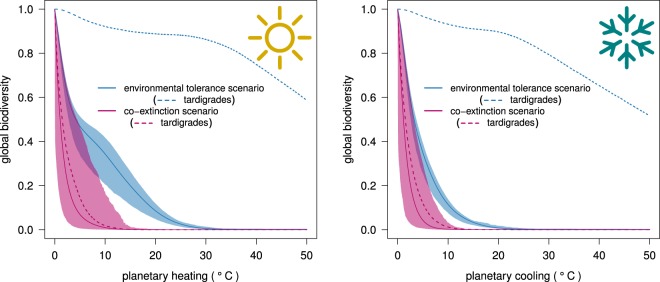
Co-extinctions reduce the robustness of planetary life to catastrophe. Response of global diversity to environmental change: progressive, monotonic increase (‘planetary heating’; left panel) or decrease (‘planetary cooling’; right panel) trajectories in local temperature. Species either go extinct based only on their tolerance to environmental conditions (‘environmental tolerance’ scenarios = blue curves), or where species go extinct not only when unable to cope with changed environmental conditions, but also following the depletion of their essential resources (‘co-extinction’ scenarios = magenta curves). Solid lines represent mean values, and shaded areas indicate the system boundaries (minimum-maximum) arising from 1000 randomly parametrized models (see Methods for details). Dotted lines show the decline in ‘tardigrade’ (extremophile) species richness in the environmental tolerance (blue) and in the co-extinction scenario (magenta) for both temperature trajectories.

The scenario curves depicting the diversity decline in the two temperature-change trajectories show how not accounting for co-extinction processes provides an unrealistic, and exceedingly optimistic perspective on the persistence of species diversity in natural communities (Fig. 1). In Fig. 1, we report the minimum and maximum recorded species diversities over all simulations, showing how the most severe cases of diversity loss are not in fact much different from the mildest ones.

Sloan and colleagues^14^ proposed to use the tolerance limits of the most resistant species to quantify life’s resilience on our planet. A more informative measure is in the area under the curve of global diversity survival *versus* environmental change (consistent with measures of ecosystem robustness to the loss of diversity^16,17^). When this measure is used to compare the two different outcomes of species diversity, it becomes easier to quantify the relative underestimation of the robustness of planetary life resulting when overlooking co-extinctions. In the heating trajectory in particular, not accounting for co-extinctions led to an underestimation of robustness varying from 61 to 1442% (median 403%) across the wide range of model settings we applied. In the cooling trajectory, the underestimation was more attenuated, but still large, and ranged from 30 to 934% (median 222%).

The differences in robustness overestimation originate from the different patterns of primary (i.e., climate-change driven) extinctions in the global heating and cooling trajectories (due, in turn, to asymmetries in species tolerance limits to low and high temperatures), with the cooling scenario projecting a much steeper decline in species diversity than the heating one (Fig. 1).

Combined with the similar curve of diversity loss obtained when co-extinctions were also taken into account, the differences in the primary extinction trajectories suggest that local food webs had different robustness to either climate heating or cooling. We tested this hypothesis by ‘disassembling’ a random sample of 1000 food webs from all simulations by removing species progressively from the least to the most tolerant to warm or cold temperatures. After each species removal, we modelled co-extinctions (in the same way as we did in our main simulations), and we kept track of the remaining diversity. The resulting curves, depicting the (co-extinction driven) decline of local diversity following direct species removal, provide an intuitive measure of network robustness to heating and cooling. In addition, we identified approximate upper and lower boundaries for robustness, obtained by removing species in decreasing and increasing order of their contribution to network persistence (that we quantified as the number of each species’ associated resources), and a reference curve obtained by removing species in random order.

**Figure 2:**
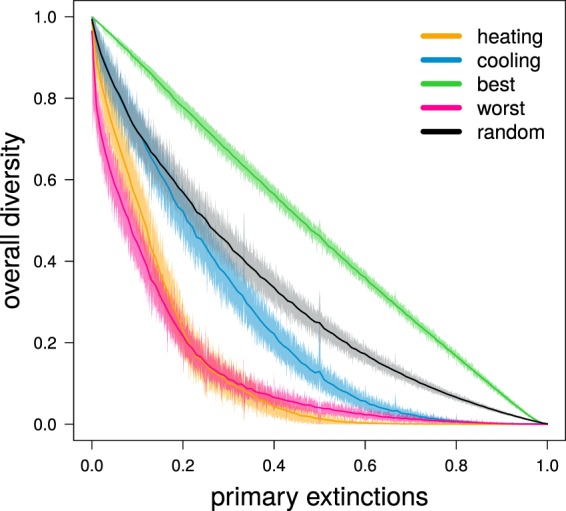
Simulated food webs are more robust to global cooling than to heating. We evaluated robustness by ‘disassembling’ a random sample of 1000 food webs. Disassembly consisted of removing species progressively from the least to the most tolerant to warm (‘heating’) or cold (‘cooling’) temperatures. We simulated co-extinctions after each species removal, and then plotted the curves depicting the (co-extinction driven) decline of local diversity following direct species removal. To obtain approximate upper and lower boundaries of robustness, we did two additional disassembly simulations for each food web by removing species in increasing (‘best’) or decreasing (‘worst’) order of their expected contribution to network persistence (measured as the number of associated resources per species). For each food web, we also obtained a reference curve by removing species in random order (‘random’). Solid lines represent mean values, while shaded areas indicate 99% confidence intervals.

As expected, we found that these resultant food webs were more robust to cooling than to heating (Fig. 2). In both cases, food-web robustness was worse than that depicted by the random reference curve, and much closer to the lower robustness boundary than to the upper one. This result is also consistent with the emerging evidence of Late Quaternary extinctions exacerbated by rapid heating, but not cooling, events^18,19^. This implies an additional warning for the future of biodiversity, because the robustness of simulated food webs to heating was largely consistent with the lower boundary obtained by removing resources in decreasing order of their importance for consumer persistence. In other words, at least in our simulations, heating tends to remove the ecologically most important species first.

This result arises because of the differences in the distributions of upper and lower temperature-tolerance limits in plants and animals. In our dataset, the lower and upper tolerance limits in plants were 1.3 ± 4.7 and 23.7 ± 4.8 °C (median ± 1*σ*), respectively, while in animals they were 23.4 ± 12.2 and 35.0 ± 4.8 °C, respectively. The overall greater tolerance to higher compared to lower temperatures in both plants and animals explains the faster global diversity loss due to environmental change observed in the cooling compared to global heating (i.e., the blue curves in Fig. 1). Furthermore, the relative difference between plants and animal adaptation to low temperatures is much larger than their relative difference in adaptation to high temperatures. In the cooling scenario, this means that when cold conditions have driven most animals to extinction, many plant species still persist; conversely, global heating will drive plants to extinction before many animals exceed their upper tolerance limits.

Increasing initial global diversity, as well as the number of communities and populated localities, raised robustness in the environmental-tolerance scenario (Figs S1, S2). This is not surprising, considering that at least in our model, both global diversity and abundance in populated localities are expected to be positively correlated with species’ ranges (see Methods). However, initial global diversity had a negligible effect on robustness in the co-extinction scenario (both for the heating and cooling trajectories; Figs. S1, S2).

In our model we implemented two separate mechanisms by which species can move from one locality to another: (*i*) a ‘species dispersal/colonization’ mechanism (Section 8, Methods), to which we are referring below, and (*ii*) a mechanism of ‘community rescue via exogenous recruitment’ (Section 9 in Methods). While the latter mechanism is capable of delaying local extinction events, it cannot alter community structure (at least qualitatively); on the contrary, the first mechanism can lead to interesting effects on both local and global species diversity.

The outcome of a colonization attempt is determined by the colonizer’s trophic level, its functional similarity to local species, and its degree of tolerance to local environmental conditions. Thus, a colonizer successfully establishes itself in a new area if better adapted to local temperature range than resident competitors when those are present, or if well adapted to local temperature range in the absence of competitors (with the additional constraint, in the co-extinction scenario, of being able to find a place in the local food web). Furthermore, the likelihood of colonization success is also limited by a measure of ‘invasion susceptibility’, which takes into account local diversity in the environmental change scenario, and network structure in the co-extinction scenario. Even if measured at the community level, invasion susceptibility partially captures the idea that resident species become more adapted to the multi-faceted ecological and environmental aspects of their home locality (besides basic climatic features such as temperature extremes) as the community becomes more diverse, and its structure becomes more complex, possibly increasing its resistance to biological invasions^20^.

The number of preliminary dispersal and colonization events (done before simulating the two trajectories of temperature change) had no effect on planetary robustness in both the environmental tolerance and the co-extinction scenarios (Figs S3, S4). Conversely, in both the global heating and cooling trajectories, increasing the amount of dispersal and colonization during climate change had a detrimental effect on robustness in the environmental tolerance scenario — increased biodiversity loss due to primary (i.e., climate-driven) extinctions only. This highlights the important role of biotic homogenization as an additional driver (and possibly, accelerator) of biodiversity loss^21^.

As we explain in the Methods, a successful colonization event can either (*a*) increase local diversity in cases where the colonizer replaces ≥ 1 local species in only some of their resource-consumer interactions without driving them to extinction, (*b*) leave local diversity as is when the colonizer drives one local species to extinction, or (*c*) reduces diversity when the colonizer outcompetes > 1 local species. Our results show that the third case was the most common in our simulations, with frequent events where strong colonizers establishing themselves in new areas had detrimental effects on local diversity. Clearly, when a species expands its geographic range via successful colonization, it also gains new opportunities for dispersal/colonization. Therefore, it is intuitive that generalist species with broad thermal niches and strong competitive ability can spread easily throughout the virtual Earth, while driving to local extinction several weaker competitors. This explains the patterns in Figs. S3 and S4, and is a phenomenon consistent with what is commonly observed in real-world systems facing the daunting problem of biological invasions^2,21^.

However, the likelihood of dispersal and colonization during environmental change had no effect on robustness in the co-extinction scenario, nor on the ratio of robustness between the two scenarios. This suggests that the speed and the magnitude of co-extinctions overshadow the relatively smaller effect of diversity loss caused by biological invasions, and that co-extinctions are likely a much more important driver of global biodiversity decline than biotic homogenization.

Neither did species adaptability to change affect robustness in either scenario, nor could it decelerate overall diversity loss (Figs. S3, S4). This latter result is clearly bound to the selected range of probabilities for local population adaptation to novel conditions. Although arbitrary, we considered the selected probabilities (ranging from 0 [no adaptability] to 0.0001) to be conservative (i.e., ensuring high adaptability in the system). In fact, the adaptability probability at each step of the simulation corresponds to the fraction of populations gaining greater thermal tolerance to changing conditions. Given the large number of starting populations in each virtual Earth (on average 31781 ± 16248 S.D.), and the high number of steps per simulation (5000, with an average global temperature change of ~ 1 °C every 100 steps), the expected number of adaptation events throughout one model run is not negligible (even if declining with time/diversity loss, which makes ecological sense).

Not surprisingly, much of the variability in the outcomes of our simulations arises from the choice of the minimum amount of resource loss capable of triggering co-extinctions in consumers (co-extinction threshold), while the plasticity of food webs towards resource reallocation (i.e., the reallocation ratio in the co-extinction model) had a minor impact on global patterns of diversity loss (Fig. S5). In our simulations we let the co-extinction threshold vary randomly between 0 and 1, where in the extreme scenario of 0 a consumer goes extinct following even a minimal loss in its resources, thus leading to pessimistic scenarios of diversity loss overall. The opposite extreme of 1 means that a consumer goes extinct only after complete depletion of its resources, depicting an opposite, overly optimistic scenario^12^. However, even under the latter, unrealistic assumption, the differences in robustness of planetary life with and without co-extinctions held firm.

Our modelling framework also offers a unique opportunity to benchmark the fascinating hypothesis that tardigrades could survive an astrophysical catastrophe^14^. In response, we explicitly included different species of tardigrade-like extremophiles in our simulations by assigning them a broad temperature tolerance^22,23^, and embedding them within trophic webs as grazers and micro-predators^24^. Despite their remarkable resistance to environmental change slowing their decline, our tardigrade-like species still could not survive co-extinctions. In fact, the transition from the state of complete tardigrade persistence to their complete extinction (in the co-extinction scenario) was abrupt, and happened far from their tolerance limits, and close to global diversity collapse (around 5 °C of heating or cooling; Fig. 1). This suggests that environmental change could promote simultaneous collapses in trophic guilds when they reach critical thresholds of environmental change. When these critical environmental conditions are breached, even the most resilient organisms are still susceptible to rapid extinction because they depend, in part, on the presence of and interactions among many other species. This highlights the real danger of modelling future diversity loss by focusing on the tolerances of single species in isolation from ecological theory^25^.

Of course, our model is an exceptionally simplified representation of ecological reality, for it would be impossible to model all species’ interactions on the planet. Nevertheless, despite its simplicity, our model yielded results consistent with real-world phenomena. For example, the near-annihilation of planetary life recorded in the end-Permian extinction event was associated with a ~ 6 °C increase in global average temperature following volcanic eruptions^26^. Ignoring for a moment the obvious differences with present fauna and flora (that we used as a reference for assigning ecologically plausible tolerance limits to our virtual species), a temperature increase of a similar magnitude would be just enough for a co-extinction-driven collapse of global biodiversity based on our simulations (Fig. 1).

In the case of the cooling trajectory, our results are also realistic compared to the global cooling event following the Chicxulub asteroid impact. The latest reconstructions estimate that the impact would have caused a 16 °C average drop in global surface temperature within three years (with at least 15 years needed to return to pre-impact temperatures)^27^. According to our projections, such a decrease in temperature would be three times larger the one needed to doom planetary life through co-extinction processes (Fig. 1b). On the one hand, this leaves little doubt about the main processes driving the extinction of the dinosaurs, irrespective of their different thermoregulation strategies^28^, because the large drop in temperature alone would have been enough to wipe out both endo- and ectotherms alike. On the other hand, that other taxa obviously survived the Chicxulub-induced nuclear winter highlights an important difference between our model and the real world. Our model parameterized a relatively homogeneous change in temperature across the virtual Earth landscape (with only a slight adjustment for faster changes at the highest latitudes that emulate current patterns in global heating). In contrast, Late Cretaceous Earth experienced a heterogeneous distribution in temperature changes (see Fig. 5 in Bardeen *et al*.^27^), explaining how some species survived by exploiting sparsely available climatic refugia. While exploring how spatial heterogeneity in climate change affects extinction patterns and processes at the global scale is beyond the scope of this study, it is a fruitful vein of inquiry for future applications and modifications of our modelling framework.

In fact, it might be possible to increase our model’s complexity by explicitly implementing population dynamics, or multiple species niche dimensions, but adding complexity would add new assumptions, and complicate interpretation. Regardless of all its potential limitations, our parsimonious exercise clearly highlights the large effect of co-extinctions on the overall robustness of planetary life to extreme environmental change, and reinforces the now well-established notion that minimizing biodiversity loss is the best approach to prevent environmental collapse.

When referring to the robustness of planetary life, we are not necessarily referring to the complete sterilization of the Earth. As also noted by Sloan *et al*.^14^, even in case of astronomical catastrophes such as the unavoidable death of the Sun, life could survive in peculiar habitats such as hydrothermal vents, and a rogue, seemingly desert Earth wandering across the Universe could still have some tiny chance of blooming again under some lucky — and unlikely — circumstances. In fact, many bacteria are even more resilient than tardigrades^29,30^, and would require much more than a Chicxulub-like impact to annihilate them. Yet, this is not reassuring news for extant metazoans, especially when compared to the outcomes of our simulations. Instead, we hope that our results provide additional confirmation that whenever a species leaves our planet, we lose much more than just a name on a list.

## Methods

We did two sets of 1000 simulations each where we first populated an ecologically plausible ‘virtual Earth’ with open, trophically structured communities that we then subjected to rapid and catastrophic global environmental-change trajectories, consisting of either a planetary heating (for example, following volcanic eruptions^26^), or cooling (as in a ‘nuclear winter’^15,27^). For each trajectory, we replicated the environmental-change phase using two alternative scenarios. In the first, we assumed that species go extinct only due to environmental tolerances being exceeded, i.e., when environmental conditions become unsuitable for their survival. In the second scenario, we considered the additional contribution of co-extinctions to diversity loss, which we modelled as extinction cascades through simulated food webs, triggered by the primary extinctions driven by environmental tolerances alone (as in the first scenario). Henceforth, we refer to those two scenarios as the ‘environmental tolerance’ and ‘co-extinction’ scenarios, respectively. By keeping track of global diversity at each step of the temperature trajectories in both scenarios, we were able to obtain two measures of robustness of planetary life (with respect to each scenario’s assumptions) as the area under the curve of diversity versus time (temperature). Comparing those measures allowed us to disentangle the direct effect of environmental tolerances from that of co-extinctions, and hence obtain, for the first time, a quantitative assessment of the relative contribution of co-extinctions to the global biodiversity crisis. We provide details on the different model components in the following sections. All the code implementing our model and permitting full replication of the study (with line-by-line detailed comments) is is freely available at https://github.com/giovannistrona/co-extinctions.

### Overview

We ran two sets of 1000 simulations for planetary heating and cooling trajectories, respectively. For each simulation, we generated a set of 10000 species having ecologically plausible tolerance limits, trophic level, and specificity in the use of resources (see *Data calibration*). We also generated a subset of 100 species as extremophiles, with large tolerance limits (see *Generating virtual species*) — we refer to those species as ‘tardigrades’, and we included them in our simulations to test explicitly how much assuming species’ tolerances to extreme conditions as a measure of their extinction risk in a changing environment^14,25^ departs from ecological realism. We attributed to each species a phenotype consisting of a set of functional traits determining a consumer’s accessibility to a given resource (see *Functional traits*). Those contributed much to the ecological realism of our simulations, playing a fundamental role in the construction of food webs (see *Building local networks*), and in the dispersal and colonization processes (see *Species dispersal/colonization*).

In each simulation, we generated a random, spatially explicit set of localities having realistic climatic features consistent with their relative geographic position on the virtual Earth. We then extracted random samples of species from the 10000 set and ‘dropped’ them into each locality, retaining in each only those species having compatibility with the local (locality) climate conditions (see *Generating virtual localities and populating them with communities*). The size of the samples of candidate species was the same for each locality, but varied among simulations, being randomly sampled with uniform probability between 100 and 1000. This provided variation in local/global diversity, which enabled us to explore the effect of varying species richness on community robustness to environmental change across simulations. We then arranged each initial pool of species in a structured food web (Section 7), and finally filtered them by excluding all species without trophic links (Section 6). To increase the ecological and biogeographical realism of the virtual Earth, we simulated a large number of dispersal events/colonization attempts, randomly sampling for each simulation with uniform probability between 10^3^ and 10^5^ events.

After the preliminary dispersal/colonization phase, we subjected the virtual Earth to ‘rapid’ global environmental change. We did not focus on a precise time scale for change, we instead assumed this to be much smaller than an evolutionary time scale by excluding speciation processes from our simulations. We focused instead on the magnitude of the temperature change as our temporal frame of reference. We modelled this in two opposing ways as a progressive, linear monotonic (1) increase, and (2) decrease in temperature. In particular, we shifted the upper and lower temperature limits of each locality by a random value of increase or decrease of temperature (in °C) sampled from a normal distribution with mean 0.01 and standard deviation 0.0025 for 5000 steps (with a final average global increase/decrease of ~ 50 °C that would be enough to annihilate all life directly, with the possible exception of some extremophile species). We also took into account a potential latitudinal effect on the speed of environmental change, as currently observed with current global warming where a nearly linear increase in temperature change has been observed in the last decades from 60° latitude upward, leading to a twofold increase in temperatures at the North Pole relative to the Equator^31^. For this, we increased the magnitude of temperature change at latitudes (*y*) > 60° north/south as (*y*-60)/30.

At each step of the environmental-change trajectory, we removed from each locality all species with temperature-tolerance limits no longer compatible with the changed conditions (see *Measuring environmental compatibility*). This single mechanism defined species loss in the environmental-tolerance scenario. In the co-extinction scenario, in addition to the primary extinctions caused by climate change at each step, we also accounted for the loss of consumers driven to extinction by the depletion of their resources. In so doing, we explored various assumptions regarding the minimum amount of resources ensuring the survival of a consumer, and the ability of the food web to rearrange interactions following species loss (see *Modelling co-extinctions*).

### Data calibration

We derived a proxy for the distribution of trophic levels in natural systems by merging into a single list all the trophic levels of individual members of a large set of food webs^32^. We computed trophic level for each species as the shortest path length (i.e., steps through network links) from the target species to a basal resource^33^. We also associated a ‘specificity’ to each individual’s trophic level, computed as the fraction of resources consumed by the target species over the total number of items in the food web to which it belonged. We obtained information on species tolerances to temperature limits from various datasets (we focused on terrestrial species — see Results and Discussion for justification). In particular, we used data for 458 species of endotherms^34^, and 239 species of terrestrial ectotherms^35^. For plants, we could only find data for cold tolerance, but not for upper temperature limits; thus, we generated an original dataset of plant temperature tolerances based on species distribution. For this, we extracted plant occurrence data from the Global Biodiversity Information Facility^36^, limiting our search to records from “observation” or “literature occurrence”. This provided > 7.2 million occurrence records of > 25000 plant species. We then restricted our search to species having > 100 occurrences (on land), which reduced the set to 4445 species. We then combined these occurrences with *Worldclim* data^37^ to identify tolerance limits. To limit the number of outlier errors, we took the lower 95% bootstrapped confidence bounds of the minimum temperatures of the coldest month across all occurrence points, and the upper 95% confidence bounds of the maximum temperatures of the hottest month across all occurrence points, as the lower and upper tolerance limits, respectively. The purpose of the total list is not to provide species-specific tolerances; rather it provides a proxy for the ‘natural’ distribution of temperature tolerances in plants at the global scale.

### Generating virtual species

In each simulation, we generated a species pool (100000 different species) to populate localities. For this, we:

1. extracted an element from the list of trophic levels/specificities;
2. if the extracted trophic level = 0 (i.e., indicating a basal resource not consuming any other species in the food web), combined that value with the tolerance limits of a randomly extracted item from the Global Biodiversity Information Facility/*Worldclim*-derived plant dataset;
3. if the extracted trophic level > 0, combined it with the tolerance limits of a randomly extracted item from the endotherm list with a probability = 0.001, and with an item extracted at random from the ectotherm list with a probability = 0.999 (taking into account the predicted difference of around three orders of magnitude between endo- and ectotherm species diversity at the global scale^38^).

For 0.1% of the species (i.e., 100), we used different, ‘tardigrade-like’ tolerance limits: a random integer between −50 and −80, and a random integer between 50 and 100 for the planetary cooling and heating) trajectories, respectively^22,23^. We attributed to these virtual tardigrades a random trophic level between 1 and 2, consistent with tardigrades’ role as grazers and micro-predators in natural systems^24^, and a corresponding specificity value (see previous section). To each non-basal species (i.e., those with trophic level > 0), we assigned a real number sampled with uniform probability from 0 to 1 to indicate the relative position of the species within its trophic level; for example, this permits an apex predator to consume another apex predator. Finally, we provided each species with a set of functional traits, defining its ‘phenotype’, that we used to assess the accessibility of a given consumer to a candidate resource, as well as functional similarity between species (see next section).

### Functional traits

The attribution of a functional phenotype to species in the simulations play an important role both to arrange species into food webs, as well as in species’ colonization processes. For each simulation, we implemented the functional traits and the related ecological mechanisms according to the following procedure:

1. We first defined an arbitrary set of 26 different functional traits, with each trait being identified by a letter from A to Z.
2. We then populated an adjacency matrix with rows and columns corresponding to functional traits with real numbers sampled uniformly from −1 to 1. The value in each cell *x*_*ij*_ indicates the degree to which the *i*^th^ trait enables a consumer to use a resource having the *j*^th^ trait. Positive values indicate that having the trait *i* increases the consumer’s accessibility to the resource with trait *j*, while negative values indicate that having the trait *j* protects (to some extent) the candidate resource from being consumed by a species with trait *i*.
3. We attributed a random set of letters of a size sampled with uniform probability from 1 to 9 to each species in the global diversity pool, as a proxy for phenotype.
4. We then quantified the ability of a consumer to use a potential resource (*ca*_*ij*_) by summing all the *x*_*ij*_ entries in the adjacency matrix for each *i*^th^ trait of the consumer and each *j*^th^ trait of the candidate resource. We rescaled this value from 0 to 1 by applying the formula:

$$\bar{c{a}_{ij}}=1-(c{a}_{max}-c{a}_{ij})/(c{a}_{max}-c{a}_{min})$$

with *ca*_min_ and *ca*_max_ being estimates of the minimum and maximum possible accessibility of a consumer to a resource. Since those values depend on the trait adjacency matrix, they are estimated as the minimum and maximum observed *ca*_*ij*_ value over a large number (1 million) of pairwise comparisons between randomly generated phenotypes.

### Measuring environmental compatibility

To assign species to a given locality (while populating the virtual Earth at the beginning of each simulation), and to determine the likelihood of success of colonization attempts as well as that of local species’ extinction events following environmental change, we focused on the compatibility between a species’ lower and upper thermal tolerance limits (sp_t, sp_T), and minimum and maximum temperatures of the target locality (*loc_t*, *loc_T*). In particular, we computed the minimum distance between the species’ tolerance limits and the local condition as:

$$Tol\_d=min[(loc\_t-sp\_t),(sp\_T-loc\_T)]$$

We then set the extinction probability to 0 in cases where the *Tol_d* value was ≥ 5, and to 1 in cases where *Tol_d* was < 0. Otherwise, we set the extinction probability to 1/(1 + *Tol_d*). Then, we evaluated the probability of a species going extinct at a given moment in the simulation by integrating over the curve of extinction probability *versus* time. We obtained this by computing *Tol_d* at 100 equally spaced time steps between time 0 and the target time, according to the corresponding temperature observed in the locality at each step. Time 0 corresponds to the moment a species established itself in the target locality, i.e., either the beginning of the environmental change simulation for native species, or the time of successful colonization for alien species. To derive the environmental conditions at a given moment (needed to compute the extinction probability at that moment, and hence compute the area under the curve), we invoked a linear relationship between time and temperature change as a good approximation, with a 1 °C of change corresponding to 1 time unit.

### Generating virtual localities and populating them with communities

We collated minimum and maximum annual temperature data from *Worldclim*^37^, and interpolated them at a resolution of 1° × 1° latitude/longitude. Then we used the list of virtual species to ‘populate’ a set of localities extracted at random from all 1° × 1° *Worldclim* cells. For each target locality, we sampled at random *n* species from the species pool, with n for each virtual Earth generated being a random integer sampled with uniform probability in [100, 1000]. This gave different outcomes of average local species richness, and hence explores the sensitivity of our results to varying species diversity (see Figs S1, S2). Among the *n* candidates, we assigned species to the target locality on the basis of their niche compatibility with local climatic conditions (see previous section).

If fewer than 5 species among the candidates were attributed to a target locality, or if basal species contributed to < 20% of local diversity, we discarded the locality and replaced it with another random locality. Otherwise, we attempted to arrange species in the pool in a structured food web (see next section for details). If this was not possible, we discarded the locality, and replaced it with another candidate one. Otherwise, after we generated the food web, we excluded all the species not having trophic links from the local species pool. These theoretical communities allowed us to test the relevance of co-extinction processes in biodiversity loss.

We reiterated the procedure until we populated a random target number of localities sampled (for each simulation) with a uniform probability between 100 and 500. As in the case of global diversity, this allowed us to test the sensitivity of our results not only with respect to sample size, but also to various spatially explicit processes we included in our model, which are affected in turn by pairwise distance between localities, and hence by the density of localities in our simulated planet.

### Building local networks

We used trophic levels (*TL*) and specificities (see *Data calibration*) to build a food web for each locality. Here, we assumed all *i* species in the target locality were potential consumers of all *j* species in the locality (with *i* ≠ *j*, and the order of the *j*^th^ species randomized for every *i*^th^ species). We then assigned species *j* as a resource to consumer *i* if *TL*_*j*_ < *TL*_*i*_ < (*TL*_*j*_ + 1) and with a probability given by trait compatibility between the target consumer and the candidate resource $(\overline{c{a}_{ij}})$. For each target consumer, we stopped the comparisons with potential resources when the number of assigned links was equal to the product of the consumer’s specificity and the total number of species in the locality (in the following, we refer to this as the ‘expected number of resources’).

In cases where the expected number of resources was not associated to a target consumer after this first step, we attempted to connect the consumer to each one of the species within the same trophic level having a smaller intra-level trophic score (see *Generating virtual species*), again with a probability dictated by resource-consumer trait compatibility $(\overline{c{a}_{ij}})$. We presented candidate resources to a target consumer in random order, with the process terminating in cases where the expected number of resources was reached. If even this second step did not result in the expected number of resources to a target consumer, and the consumer belonged to a trophic level > 1, we attempted to link a target consumer to each one of the resources of two trophic levels lower. As in the previous steps, we established links with a probability given by resource-consumer trait compatibility, presented potential resources to target consumer in random order, and terminated the process when the maximum number of expected resources was reached. This three-step procedure gives more realism to the food webs by increasing their connectivity and clustering, and by reducing their average path length.

Once all species in the locality were evaluated as potential consumers, we identified the set of basal species as those with a trophic level = 0. We then filtered the network by excluding from each target locality all species for which a path to basal species did not exist. This prevented the generation of unrealistic food webs where, for example, large carnivores have access to herbivore prey, but the latter have no access to food. Finally, we assigned weights to the food-web links on the basis of functional-trait compatibility between the target resource-consumer pair $(\overline{c{a}_{ij}})$.

### Species dispersal/colonization

To make the virtual communities connected within a biogeographical context, we implemented a mechanism of species dispersal/colonization from one locality to another (with slight differences between the environmental-tolerance and co-extinction scenarios — see below). We assumed that the likelihood of successful colonization is a function of the distance between two localities, of the structure of the target community (with more mature/structured communities being less open to colonization than degraded communities), and of the ability of the colonizer to establish itself in the new locality. The latter depends on several factors, particularly the degree of tolerance of the colonizer to local climatic conditions, the colonizer’s trophic level, and the colonizer’s functional traits.

We simulated a single dispersal/colonization attempt in the model as follows:

1. A random pair of localities *i* and *j* (with *i* ≠ *j*) is sampled with uniform probability;
2. A random species is then sampled with uniform probability from the source locality *i*; this disperses successfully to locality *j* with probability *d*_*ij*_^−1^, with *d*_*ij*_ representing the shortest Euclidean distance (*d*) between two localities;
3. In cases of successful dispersal, colonization is attempted. Colonization fails regardless of the colonizer’s identity with a probability given by 1 - *p*_*i*_ (the target locality’s invasion susceptibility), which is equal to the connectance of the local food web *in the co-extinction scenario* (i.e., the ratio between the number of links in a food web to its squared number of nodes, with the latter being the theoretical maximum number of possible pairwise interactions)^20^, and to the ratio of initial diversity (i.e., the number of species present in the target locality at the beginning of the simulation) to the diversity at the time of the colonization attempt *in the tolerance scenario*.
4. In the *environmental-tolerance* scenario, the colonizer outcompetes species of the same trophic level that are less tolerant to local environmental conditions (see Section 5), with a probability given by their trait similarity. We calculated this probability as 2.0 × M/T, where T is the total number of traits in both species’ phenotypes, and M is the number of matches (this formula yields 1 if the sequences are identical, and 0 if they have nothing in common). Thus, trait similarity identifies the potential invader’s competitors, and then colonization succeeds only if the invader is better adapted to the novel, rapidly changing conditions than local species. We acknowledge that ‘adaptation’ here is limited to thermal tolerance, and that there could be several other ecological aspects making a native species ‘better’ adapted to its native environment than an alien one, but in our simplified world, thermal tolerance was in fact the main element determining species distribution, making our choice ecologically reasonable. In cases where the potential colonizer has no competitors in the target locality, it succeeds in colonization with a probability given by its compatibility with local environmental conditions. That is, invaders with no local competitors succeed in colonization with the only constraint of tolerance to local temperature range. This completes the explanation for the colonization mechanism in the environmental-tolerance scenario; all the following steps in this section refer to the co-extinction scenario only.
5. In the *co-extinction* scenario, if colonization has not failed due to local invasion susceptibility alone, the potential colonizer attempts to ‘enter’ the food web. If the colonizer is not a basal resource (i.e., trophic level > 1), it will need to find some resources to consume in order to establish itself in the target locality. For this, it will attempt to replace each local consumer having the same trophic level in each one of its resource-consumer interactions. For this, for each link in the network involving a consumer of the same trophic level as the potential colonizer, the local consumer will be replaced by the colonizer if the colonizer has an identical or better compatibility with local environmental conditions than the local species, and with a probability given by the rescaled trait compatibility $(\overline{c{a}_{ij}})$ between the colonizer and the target resource. In cases of successful colonization, the old trophic link is removed from the food web, while the new one between the colonizer and the local resource is added. The interaction weight is inherited from the original link between the local resource and the out-competed local consumer. This choice is motivated by the fact that the weight of a trophic link in our modelling framework represents resource availability. The availability of local resources is (at least at the beginning of the invasion) not affected by the switch in consumers between the previous species and the invader. Thus, an alien species replacing ≥ 1 local species in some or all of their trophic interactions will have access to the same amount of resources previously used by the native, now-outcompeted consumer/s, and hence be assigned trophic links having weight equal to that of the original interactions.
6. In cases where a non-basal colonizer has successfully established, the model accounts for the possibility that the colonizer becomes an additional resource for other species. Thus, for each resource consumer link in the food web, if the colonizer has the same trophic level of target resource, is accessible to consumers (i.e., with probability $\overline{c{a}_{ij}}$), and is well-adapted to local climate (i.e., with probability equal to climatic suitability), then the colonizer might be used as an additional resource. A new link is therefore added to the food web, connecting the colonizer to the target local consumer, with an interaction weight equal to a random fraction of the original link between local resource and consumer. Such a link is then maintained in the network.
7. If the potential colonizer is a basal resource, then it will have to outcompete, to some degree, another local basal resource, under the assumption that the system is at equilibrium carrying capacity. In the real world, such a process could lead to loss of local diversity, especially in cases where the local, outcompeted resource supports several species unable to survive by establishing novel interactions with the colonizer. Despite being ecologically interesting, analyzing this process further is beyond the scope of our study. To avoid situations where our results are biased by diversity loss triggered by biological invasions, we conservatively allowed basal colonizers to replace local basal species as resources in resource-consumer interactions, but we did not consider normal outcompeting events. As in the colonization processes we described above, the colonizer must have trait compatibility with the target local consumer (which also has ecological meaning, since it might indicate that the colonizer’s phenotype is in fact functionally similar to that of the local resource it is outcompeting).

According to these mechanisms, a successful colonization event can either: (*a*) lead to an increase in local diversity if the colonizer replaces ≥ 1 local species in only some of their resource-consumer interactions without fully outcompeting any of them (or, in the environmental-tolerance scenario, when a colonizer establishes itself by exploiting an empty niche), (*b*) leave local diversity unchanged even if the colonizer completely outcompetes a single local species, or (*c*) reduce diversity if the colonizer entirely outcompetes > 1 local species. In the *environmental-tolerance scenario*, diversity can also increase when a colonizer establishes itself by exploiting an empty niche. In the *co-extinction* scenario, when the colonizer is a non-basal resource that becomes an additional resource to local species, the potential increase in diversity is also paired to an increase in overall network interaction strength. However, this does not necessarily produce an increase in network connectance, since this decreases more rapidly with the addition of nodes than it increases with the addition of links.

### Community rescue via exogenous recruitment

In addition to colonization events, we also implemented an explicit mechanism of recruitment from closer localities as a means to replenish depleted resource stocks. At each step of the simulation, every target locality *i* receives exogenous recruits from any other source locality (*j*) with a probability = 0.01. When recruitment happens, the interaction strength of each link of the food web in the *i*^th^ locality is increased (up to the original interaction strength observed at the beginning of the simulation) by *d*_*ij*_^−1^ times its original value (with *d*_*ij*_ representing the shortest linear distance between two localities), if the resource species in the target link is also present in the *j*^th^ (i.e., the source) locality. As we discussed above, the interaction weight in our model is a proxy for resource availability, and hence, population size of the focal resource. The magnitude of recruitment is proportional to distance in that we expect more recruitment from closer localities than from more distant ones. The inverse relationship between distance and increase in interaction weight (ideally representing an increase in the target resource’s population size) offers a simple way to account for this aspect in our simulations.

### Species adaptability to change

Although in our simulations we assumed environmental change happens on a temporal scale much smaller than evolutionary processes, we still provided species with the ability to develop some thermal adaptation — an ability observed in nature in both invertebrates^39^ and vertebrates^40^. We assumed that in each simulation step, each species shifted its thermal limits (by a random value sampled from a normal distribution with mean 0.75 and standard deviation 0.25) with a particular probability. We sampled this probability at random (with uniform probability) between 0.0 and 0.0001, and set it at the beginning of the simulation to remain stable throughout each simulation. Using a variable probability across individual simulations allowed us to test the sensitivity of our results to overall species adaptability.

### Modelling co-extinctions

We modelled co-extinctions following Säterberg *et al*.^12^. We initiated simulations with the removal of species obliterated by mismatches of environmental tolerance as described above. This possibly triggered co-extinctions in consumers that experienced an initial loss of resources exceeding a certain fraction. We quantified the ‘amount’ of resources as the cumulative weights of the links from a target consumer to all of its resources, and we explored various thresholds for co-extinctions (in terms of fraction of lost interactions) by sampling in each simulation a real number at random (with uniform probability) between 0 and 1. We permitted the ‘rewiring’ of lost interactions; that is, we allowed resources made available by the extinction of some of their original consumers to be redistributed among extant consumers^14^. We constrained the redistribution among consumers already using the target resource, proportionally to the current use of that resource. We weighted the amount of redistributed resources by a ‘reallocation ratio’. As in the case of the co-extinction threshold, we explored different reallocation ratios by sampling in each simulation a real number at random (with uniform probability) between 0 and 1. We reiterated the three steps of the co-extinction process — (1) removing lost species, (2) evaluating lost resources, and (3) including the interaction rewiring — until there were no new (i.e., cascaded) co-extinctions, or when all species in the network had gone extinct.

### Exploring food-web robustness to environmental-change trajectories

To explore the robustness of local food webs towards either climate heating or cooling, we ‘disassembled’ 1000 food webs sampled at random between all food webs in all virtual Earths (*after* the pre-dispersal/colonization phase to use the same food webs we subjected to environmental change). Disassembly consisted of removing species progressively from the least to the most tolerant to warm or cold temperatures alternatively. Following each species removal, we modelled co-extinctions according to the same procedure we used in our main simulations (see previous section). Finally, to assess robustness of the target food web, we built curves tracking the overall decline of local species diversity (accounting for both primary extinctions triggered directly by climate change, and co-extinctions) following progressive temperature change. We also identified approximate upper and lower boundaries for robustness by replicating the same procedure under different criteria of species removal^16^. To approximate the upper boundary of food-web robustness, we progressively removed species from those supporting the lowest number of consumers to those supporting the most consumers. To approximate the lower robustness boundary, we removed species in reverse order, starting from those supporting the most consumers, and then moving progressively towards species supporting the fewest or no consumers. We also generated reference curves for each food web by removing species in random order.

## Electronic supplementary material

Supplementary Figures

## Data Availability

All the code and data ensuring full replicability of our analyses are freely available from: github.com/giovannistrona/co-extinctions.
