# Supplementary material for: Co-extinctions annihilate planetary life during extreme environmental change
Source: Sci Rep. 2018 Nov 13;8:16724. doi: 10.1038/s41598-018-35068-1 (PMC6233172; doi:10.1038/s41598-018-35068-1)
Supplement: Supplementary file 1 — Supplementary Figures [file 41598_2018_35068_MOESM1_ESM.pdf]

Supplementary Information

Co-extinctions annihilate planetary life during extreme environmental change

Giovanni Strona*[1], Corey J. A. Bradshaw[2]

[1]European Commission, Joint Research Centre, Directorate D – Sustainable Resources, Ispra, Italy. giovanni.strona@ec.europa.eu

[2]Global Ecology, College of Science and Engineering, Flinders University, Adelaide, Australia. corey.bradshaw@flinders.edu.au

*Corresponding author, to whom correspondence and material requests should be addressed.

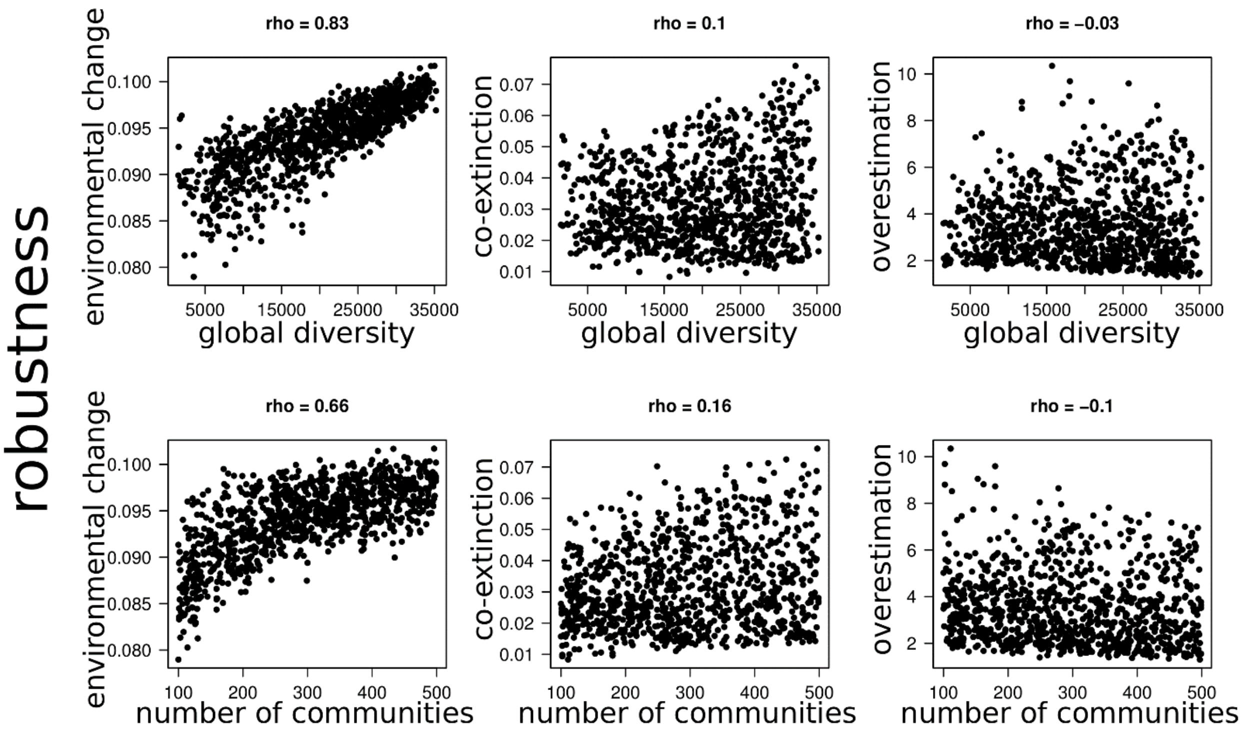

Supplementary Figure S1 | High global diversity and number of communities increase resilience to global warming in the environmental-change scenario, but not in the co-extinction scenario. Points in each panel correspond to the 1000 model simulations. In the environmental-change scenario, species go extinct only when local environmental conditions exceed their tolerance limits. In the co-extinction scenario, species go extinct not only when no longer able to survive in the local climatic conditions, but also following the depletion of their resources. Left and middle panels show the relationship between initial global diversity and the number of communities (i.e., food webs) we used in the simulations, and planetary resilience to global warming (i.e., the area under the curve of global diversity decline following extreme, progressive increase in local temperatures) in both the environmental-change and the co-extinction scenarios. Right panels show the relationships between initial diversity and number of communities *versus* resilience overestimation derived from not accounting for co-extinction processes, here quantified as the ratio between resilience measured in the environmental-change scenario and in the co-extinction scenario.

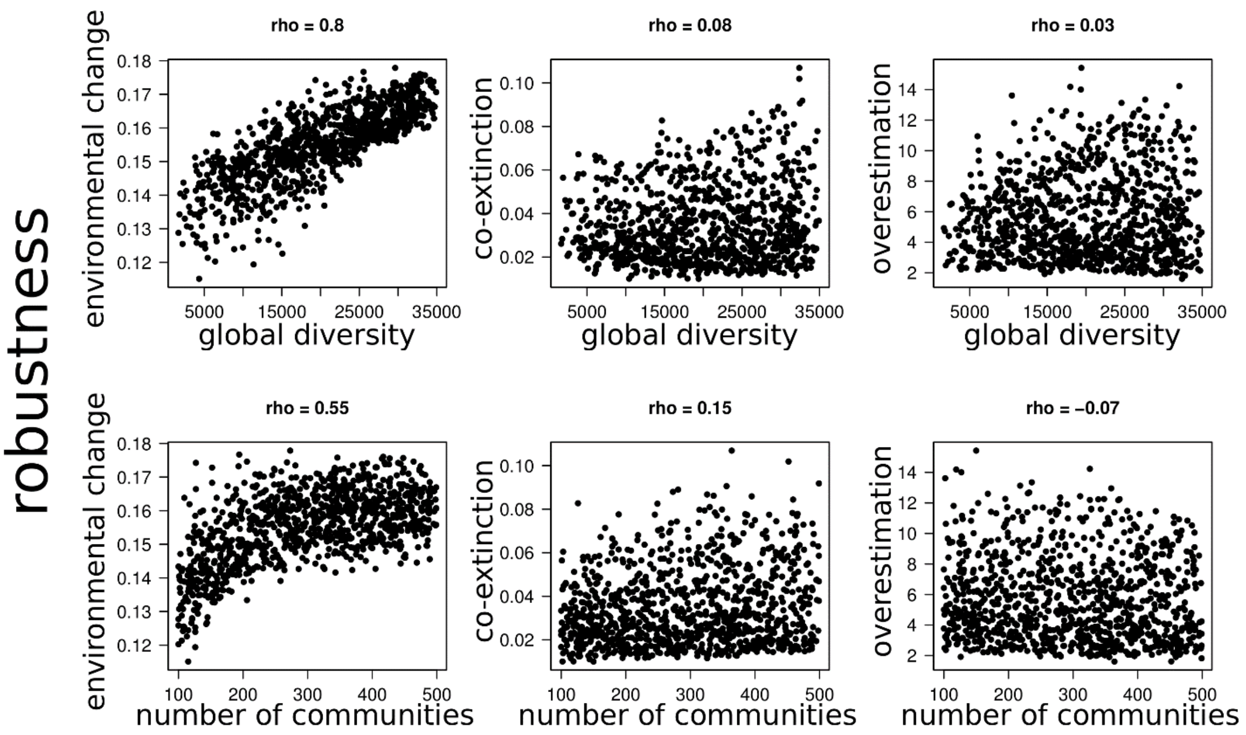

Supplementary Figure S2 | High global diversity and number of communities increase resilience to planetary cooling ('nuclear winter') in the environmental-change scenario, but not in the co-extinction scenario. Points in each panel correspond to the 1000 model simulations. In the environmental-change scenario, species go extinct only when local environmental conditions exceed their tolerance limits. In the co-extinction scenario, species go extinct not only when no longer able to survive in the local climatic conditions, but also following the depletion of their resources. Left and middle panels show the relationship between initial global diversity and the number of communities (i.e., food webs) we used in the simulations, and planetary resilience to planetary cooling (i.e., the area under the curve of global diversity decline following extreme, progressive decrease in local temperatures) in both the environmental-change and the co-extinction scenarios. Right panels show the relationships between initial diversity and number of communities *versus* resilience overestimation deriving from not accounting for co-extinction processes, here quantified as the ratio between resilience measured in the environmental change scenario and in the co-extinction scenario.

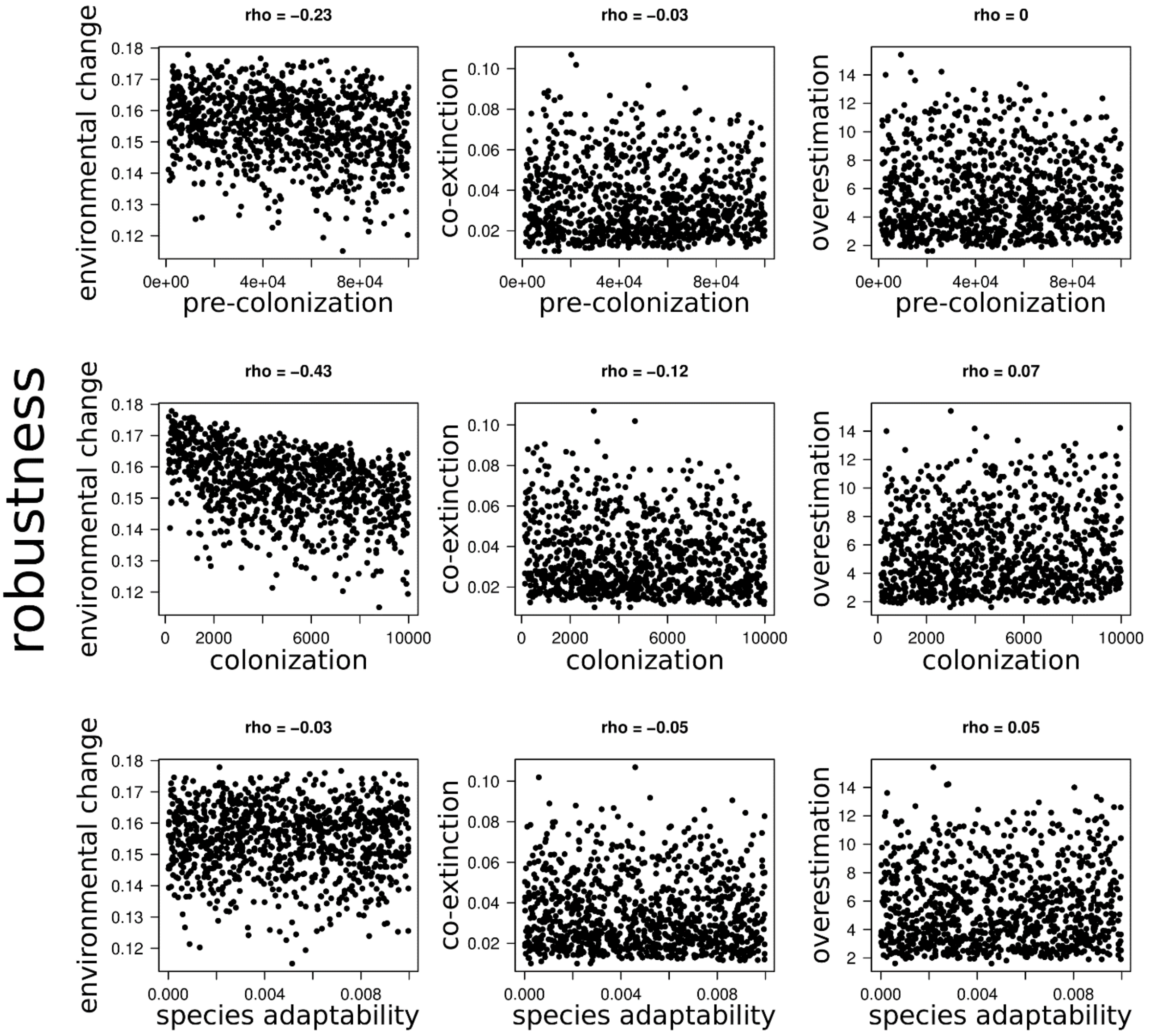

Supplementary Figure S3 | The frequency of species dispersal and colonization events (but not species thermal adaptability) affects the resilience of planetary life to global warming. Points in each panel correspond to the 1000 model simulations. In the environmental change scenario, species go extinct only when local environmental conditions exceed their tolerance limits. In the co-extinction scenario, species go extinct not only when no longer able to survive in the local climatic conditions, but also following the depletion of their resources. *y*-axes in the upper and middle panels indicate the number of dispersal/colonization attempts every 1 °C of temperature rise. *y*-axes in the lower panels indicate the frequency of species-adaptation events (see Methods in the main text) for every 1 °C of temperature rise.

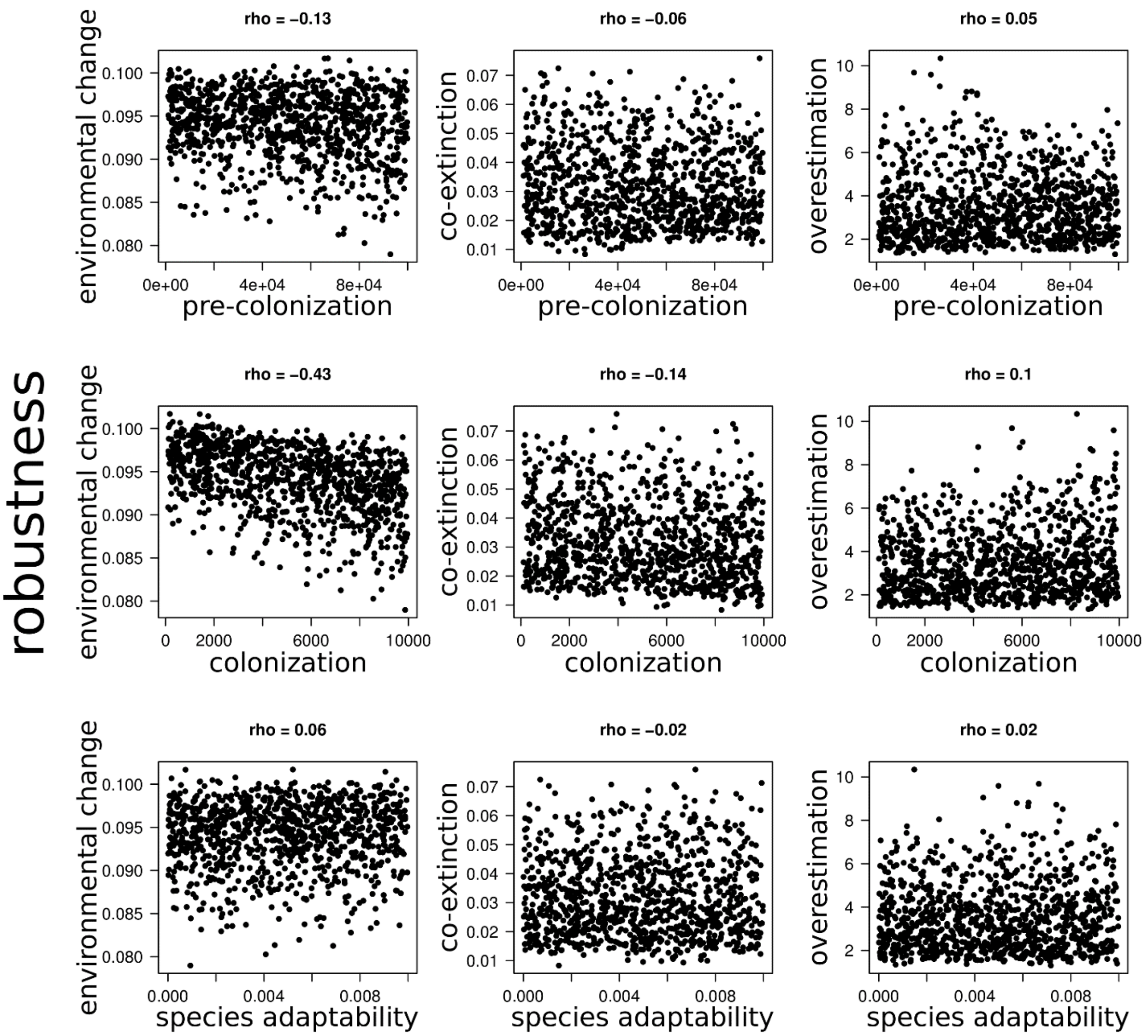

Supplementary Figure S4 | The frequency of species dispersal and colonization events (but not species thermal adaptability) affects planetary life resilience to nuclear winter. Points in each panel correspond to the 1000 model simulations. In the environmental-change scenario, species go extinct only when local environmental conditions exceed their tolerance limits. In the co-extinction scenario, species go extinct not only when no longer able to survive in the local climatic conditions, but also following the depletion of their resources. *y*-axes in the upper and middle panels indicate the number of dispersal/colonization attempts every 1 °C of temperature decline. *y*-axes in the lower panels indicate the frequency of species adaptation events (see Methods in the main text) for every 1 °C of temperature decline.

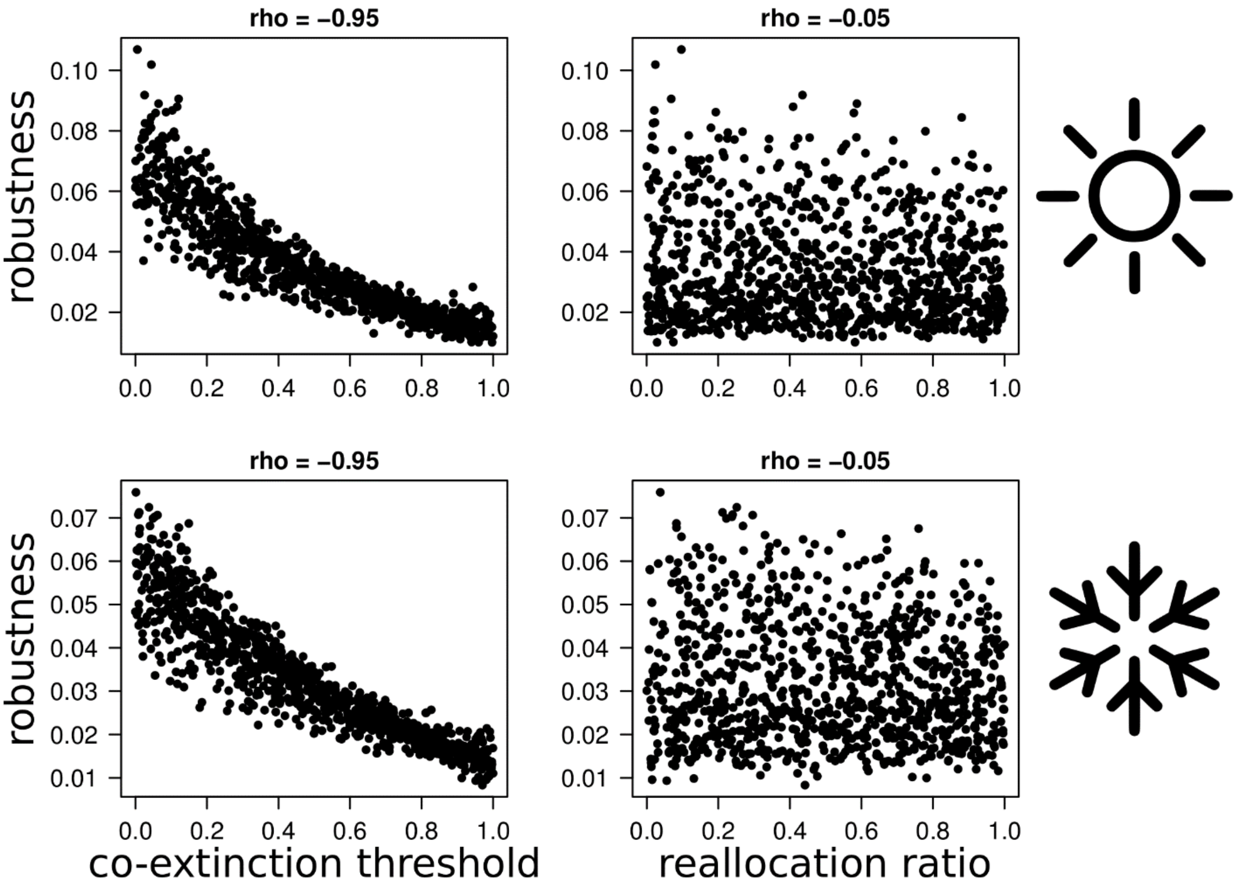

Supplementary Figure S5 | The co-extinction threshold drives the resilience of planetary life to global environmental change, while food web plasticity has no effect. Points in each panel correspond to the 1000 model simulations. The co-extinction threshold is the minimum amount of resources (computed as the fraction of the cumulative interaction weight — i.e., the sum of weights for all food-web edges connecting a resource to the target consumer — at a given moment in respect to the starting one — i.e., the cumulative weight at the beginning of the simulation). The reallocation ratio is the maximum fraction of resource becoming available after the extinction of one of their consumers that can be re-distributed among extant consumers (see Methods in the main text for details). Upper and lower panels show the results for the global warming and the nuclear winter trajectories, respectively.
